# Analysis of HIV Early Infant Diagnosis Data to Estimate Rates of Perinatal HIV Transmission in Zambia

**DOI:** 10.1371/journal.pone.0042859

**Published:** 2012-08-17

**Authors:** Kwasi Torpey, Justin Mandala, Prisca Kasonde, Gail Bryan-Mofya, Maximillian Bweupe, Jonathan Mukundu, Chilunje Zimba, Catherine Mwale, Hilary Lumano, Michael Welsh

**Affiliations:** 1 FHI 360, Durham, North Carolina, United States of America; 2 Management Sciences for Health, Cambridge, Massachusetts, United States of America; 3 Ministry of Health Zambia, Lusaka, Zambia; University of Cape Town, South Africa

## Abstract

**Background:**

Mother-to-child transmission of HIV (MTCT) remains the most prevalent source of pediatric HIV infection. Most PMTCT (prevention of mother-to-child transmission of HIV) programs have concentrated monitoring and evaluation efforts on process rather than on outcome indicators. In this paper, we review service data from 28,320 children born to HIV-positive mothers to estimate MTCT rates.

**Method:**

This study analyzed DNA PCR results and PMTCT data from perinatally exposed children zero to 12 months of age from five Zambian provinces between September 2007 and July 2010.

**Results:**

The majority of children (58.6%) had a PCR test conducted between age six weeks and six months. Exclusive breastfeeding (56.8%) was the most frequent feeding method. An estimated 45.9% of mothers were below 30 years old and 93.3% had disclosed their HIV status. In terms of ARV regimen for PMTCT, 32.7% received AZT+single dose NVP (sdNVP), 30.9% received highly active antiretroviral treatment (HAART), 19.6% received sdNVP only and 12.9% received no ARVs. Transmission rates at six weeks when ARVs were received by both mother and baby, mother only, baby only, and none were 5.8%, 10.5%, 15.8% and 21.8% respectively. Transmission rates at six weeks where mother received HAART, AZT+sd NVP, sdNVP, and no intervention were 4.2%, 6.8%, 8.7% and 20.1% respectively. Based on adjusted analysis including ARV exposures and non ARV-related parameters, lower rates of positive PCR results were associated with 1) both mother and infant receiving prophylaxis, 2) children never breastfed and 3) mother being 30 years old or greater.

Overall between September 2007 and July 2010, 12.2% of PCR results were HIV positive. Between September 2007 and January 2009, then between February 2009 and July 2010, proportions of positive PCR results were 15.1% and 11% respectively, a significant difference.

**Conclusion:**

The use of ARV drugs reduces vertical transmission of HIV in a program setting. Non-chemoprophylactic factors also play a significant role in HIV transmission. The overall change in the proportions of positive PCR results over time is more likely an indication of better PMTCT implementation. Determination of the outcomes of PMTCT in program settings is feasible but requires accurate documentation and analysis.

## Introduction

Mother-to-child transmission of HIV (MTCT) remains the most prevalent source of pediatric HIV infection. In 2010 alone, an estimated 390,000 children were infected with HIV, 90% of whom live in sub-Saharan Africa [1]. Pediatric HIV threatens to reverse gains made in controlling child mortality in African countries with high HIV seroprevalence. HIV infection accounts for more than 20% of child deaths in southern Africa compared with approximately 3% globally [2].

This threat has been recognized by the international community, which has spurred advocacy, political and financial responses to reduce – and ultimately eliminate – MTCT. Indeed in recent years the number of women accessing programs that aim to prevent mother-to-child transmission of HIV has steadily increased [3]. Most prevention of mother-to-child transmission (PMTCT) programs have concentrated monitoring and evaluation efforts on measuring process indicators such as acceptance rate of HIV testing and counseling or proportion of HIV-positive women provided with antiretroviral drugs.

However, in order to compare progress across different PMTCT approaches and to mount a coordinated response, there is need to move beyond process indicators and measure PMTCT outcomes. Many approaches – with their advantages and limitations – have been suggested to measure outcomes of PMTCT [4], [5]. One of the indicators proposed to measure PMTCT outcomes is the number of “infant infections averted” that can also be interpreted as the rate of MTCT.

In recent years, access to early infant diagnosis of HIV (EID) has substantially increased in Zambia. This programmatic success in scaling up EID provides an opportunity to measure the rate of MTCT, an outcome of PMTCT.

Earlier we published test results of 8,237 perinatally exposed children to estimate HIV transmission rates in a program setting in Zambia [6], [7]. In this paper, we provide an update to those results, analyzing data from 28,320 perinatally exposed infants, which includes the previously published data. This new analysis is also an opportunity to review how changes in the PMTCT policy and implementation has affected the rate of MTCT. Indeed, between 2007 and 2010, Zambian PMTCT programs had gradually shifted from sdNVP based to more efficacious ARVs regimens – AZT+sdNVP or HAART.

An analysis of DNA polymerase chain reaction (PCR) test results was conducted, coupled with PMTCT observational data, to estimate MTCT rates among HIV-exposed children aged zero to six weeks, six weeks to six months, and six months to 12 months. MTCT rates were estimated according to 1) who received an antiretroviral (ARV) regimen (mother, infant, both, or neither), 2) type of ARV regimen provided, 3) place of delivery, 4) mother's age, and 5) whether the mother disclosed her HIV status.

## Methods

### Study context

The Zambia Prevention Care and Treatment Partnership (ZPCT) project, launched in 2005, and its follow-on project, ZPCT II, have supported the continuum of care for PMTCT: from HIV testing of pregnant women to early diagnosis of HIV among children born to HIV-positive mothers. ZPCT support covers five of the nine provinces in Zambia, namely Central, Copperbelt, Luapula, North-Western, and Northern Provinces. Support is provided at the primary, secondary and tertiary level health facilities as well as at the community level.

PMTCT activities are implemented in 350 public and private ZPCT-supported health facilities. Women that test HIV-positive are offered a CD4 count either onsite or offsite through a sample referral system. CD4 count results are used to determine eligibility for lifelong antiretroviral treatment (ART) according to the Zambian national PMTCT guidelines [8]. Women with a CD4 count less than or equal to 350 cells/mm^3^ are offered lifelong highly active antiretroviral therapy (HAART) to be initiated as soon as possible. Those women with a CD4 count above 350 are offered zidovudine (AZT) as early as 14 weeks of pregnancy and intrapartum single dose nevirapine (sdNVP)+lamivudine (3TC). The “sdNVP only” regimen is the last option when women, for any reason, are unable to initiate AZT short course or lifelong ART.

Prior to WHO's 2010 revision of the global PMTCT recommendations, sdNVP constituted the backbone of PMTCT implemented in Zambia [9]. ZPCT does not provide free infant formula and encourages exclusive breastfeeding for the first six months of life when formula feeding is not affordable, feasible, acceptable, safe, and sustainable. The program supports early infant diagnosis (EID) of HIV and refers HIV-infected children to care and treatment centers for early initiation of pediatric ART.

In 2007, ZPCT supported the Ministry of Health to establish a DNA PCR laboratory capacity at *Arthur Davidson Children's Hospital*, a tertiary health facility located in the Copperbelt Province. Dried Blood Spot (DBS) samples are collected at PMTCT sites by trained staff and then transported periodically to a central collection point by motorcycle provided by ZPCT. At the collection points DBS packages are mailed to *Arthur Davidson Children's Hospital* using Expedited Mail System (EMS) with the Zambia Postal Services. PCR results are then sent back to PMTCT sites in the reverse direction.

EID is recommended at age 6-weeks postnatal then at ages six months and 12 months to all HIV-exposed infants. In reality, DBS samples are collected from children age six weeks to 18 months; this is partly due to the fact that EID is a relatively new component of PMTCT programs and there is still a substantial backlog of HIV-exposed children that remain to be tested. Routine PCR data benefit from strong data quality assurance and represent an important opportunity to assess MTCT rates among HIV-exposed children.

### DNA PCR testing methodology

Testing is performed on 5 mm disks of the DBS which are punched into sterile 2 mL cryovials and washed in specimen wash solution for 30 minutes twice to remove hemoglobin. Working extraction solution, a detergent solution containing proteinase K and HIV-1 internal control (IC), is then used to extract and lyse the DNA containing leucocytes from the disks. 50 uL of the extracted DNA solution is added to an equal volume of working mastermix and then amplified for 35 cycles with a final hold stage at 72° centigrade for 15 minutes at which the amplified products are denatured. Denatured amplicon is hybridized in separate HIV-1 and HIV-1 IC target specific probe coated microwell plates, washed in buffer, conjugated to Avidin-Horseradish Peroxidase, washed again and a substrate added to give a colored complex. Stop solution is added to the colored complex after 10 minutes incubation and detection is completed by colorimetric reading at 450 nm. Any value<0.2 A_450_ are considered negative, ≥0.2 A_450_ and <0.8 A_450_ are considered indeterminate and ≥0.8 A_450_ are positive. Duplicate repeat testing is performed on indeterminate specimen and results interpreted using 0.2 A_450_ as the cutoff point.

### Design

This is a descriptive observational study of HIV EID activities. This study analyzed all DNA PCR results and PMTCT data from perinatally exposed children 0 to 12 months of age seen at the *Arthur Davidson Children's Hospital*. There was no sample selection; all PCR results and PMTCT data available were considered in this analysis. Children lived from the five provinces supported by ZPCT – Central, Copperbelt, Luapula, North-Western, and Northern Provinces – between September 2007 and July 2010. This study is an update of an earlier analysis which covered the period between September 2007 and January 2009 [6], [7].

### Data collection, entry and analysis

Health care workers at maternal, neonatal and child health (MNCH) units routinely collected dry blood spots (DBS) for PCR tests for all babies that were perinatally exposed to HIV. They completed PCR requisition forms to accompany the DBS samples. Information on any PMTCT service offered – e.g. type of ARV regimen received by mother and baby, infant feeding method, disclosure of HIV status – was also recorded on the PCR requisition form. The DBS PCR requisition forms were populated with data from the mother's antenatal card and child's “under-5” cards. The client information collected included age of the child at the time of DBS collection, maternal age, feeding method, type of ARV regimen given to mother and/or baby, mode of delivery, and whether the mother disclosed her HIV-positive status to her partner.

In the PCR laboratory, a Microsoft Access database was used to store and process data from the PCR requisition forms and the PCR results. Internal accuracy and consistency of the data were regularly assessed. Electronic data were verified against paper records and cleaned as appropriate. Repeat samples were excluded from the analysis.

The MTCT rates were estimated, along with 95% confidence intervals, separately for each age group and for specific PMTCT interventions received by mothers and children. Mantel-Haenszel methods were used to study the associations between vertical transmission of HIV and chemoprophylactic and non-chemoprophylactic factors controlling for province as a stratification factor. All p-values should be interpreted as descriptive measures of association rather than as inferential statistics applicable to a larger population. A multiple logistic regression model was applied to assess the associations between vertical transmission rates and both ARV related and non-ARV related interventions and population characteristics. The model also controlled for the ARV regimen received by the mother. From this model, we estimated odds ratios along with 95% confidence intervals separately for each age group. Missing data were ignored. Analyses were performed using SAS, version 9.3 (SAS Institute, Cary, NC).

To assess changes in PMTCT implementation over time, we compared the rates of positive PCR results between 1) DBS samples collected from September 2007 to January 2009, and 2) those collected from February 2009 to July 2010. We also assessed changes in the population characteristics between these two periods.

### Ethical approval

Ethical Approval was granted by the University of Zambia Research and Ethics Committee as well as by the Protection of Human Subjects Committee of FHI 360, North Carolina, USA with final clearance from the Ministry of Health, Zambia.

## Results

### Study population

In total, the study analyzed data from 28,320 babies aged between 0 and 12 months covering the period September 2007 to July 2010. The children came from 317 facilities in 40 districts across five provinces. (Table 1). Previously published analyses included data for 8,237 of these babies [6], [7].

**Table 1:** Mother and child characteristics.

All infants up to 12 months of age	Overall period: September 2007 to July 2010		Period 1: September 2007 to January 2009		Period 2: February 2009 to July 2010	
Age at PCR blood collection	N	(%)	N	(%)	N	(%)
0–6 weeks	6,059	(21.4)	1,649	(19.0)	4,410	(22.4)
6 weeks–6 months	16,597	(58.6)	4,969	(57.4)	11,628	(59.1)
6–12 months	5,664	(20.0)	2,036	(23.5)	3,628	(18.4)
**Infant/Child sex**						
Female	14,104	(49.8)	4,380	(50.6)	9,724	(49.4)
Male	14,007	(49.5)	4,259	(49.2)	9,748	(49.5)
*Missing data*	*209*	*(0.7)*	*15*	*(0.1)*	*194*	*(0.9)*
**HIV status disclosure**						
Did not disclose	1,781	(6.3)	471	(5.4)	1,310	(6.6)
Disclosed	26,432	(93.3)	8,168	(94.3)	18,264	(92.8)
*Missing data*	*107*	*(0.4)*	*15*	*(0.1)*	*94*	*(0.4)*
**Mother Age**						
≥30 years old	6,373	(22.5)	1,933	(22.3)	4,440	(22.6)
<30 years old	12,985	(45.9)	4,100	(47.4)	8,885	(45.2)
*Missing data*	*8,972*	*(31.6)*	*2,621*	*(30.3)*	*6,341*	*(32.2)*
**Infant feeding status at PCR time**						
Never breastfed	3,833	(13.5)	1,355	(15.6)	2,478	(12.6)
Still breastfeeding	20,757	(73.3)	5,990	(69.2)	14,767	(75.0)
Stopped BF	3,420	(12.1)	1,288	(14.8)	2,132	(10.8)
*Missing data*	*310*	*(1.1)*	*21*	*(0.2)*	*289*	*(1.4)*
**Delivery types**						
In HF, normal delivery	23,233	(82.0)	7,066	(81.6)	16,167	(82.2)
In HF and C-S	1,155	(4.1)	418	(4.8)	737	(3.7)
Home	3,674	(13.0)	1,145	(13.2)	2,529	(12.8)
*Missing data*	*258*	*(0.9)*	*25*	*(0.2)*	*233*	*(1.1)*
**Who received ARVs for PMTCT**						
Mother and infant	20,083	(70.9)	6,113	(70.6)	13,970	(71.0)
Mother only	2,521	(8.9)	836	(9.6)	1,685	(8.5)
Infant only	445	(1.6)	232	(2.6)	213	(1.0)
Neither	3,012	(10.6)	1,249	(14.4)	1,763	(8.9)
*Missing data*	*2,259*	*(8.0)*	*224*	*(2.5)*	*2035*	*(10.3)*
**Mother's PMTCT ARV regimen**						
AZT+ sdNVP	9,259	(32.7)	2,123	(24.5)	7,136	(36.2)
HAART	8,761	(30.9)	2,593	(29.9)	6,168	(31.3)
sdNVP	5,540	(19.6)	2,348	(27.1)	3,192	(16.2)
None	3,653	(12.9)	1,593	(18.0)	2,090	(10.6)
*Missing data*	*1,107*	*(3.9)*	*27*	*(0.3)*	*1,080*	*(5.4)*

Half of the infants were male. The majority of children (58.6%) had a PCR test conducted between age six weeks and six months; 71.5% received ARVs prophylaxis; 82.0%were delivered in health facilities (HF) without C-section and 86.4% were ever breastfed. Of those who ever breastfed, 96.2% were still breastfeeding at age six weeks and 52.8% at age six months. Exclusive breastfeeding (56.8%) was the most frequent feeding method as reported by mothers followed by mixed feeding (22%).

The majority of mothers were below 30 years old (45.9%). Most women disclosed their HIV-positive status (93.3%). Among mothers, 32.7% received AZT+sdNVP, 30.9% received HAART, 19.6% received sdNVP only, and 12.9% did not receive ARVs.

In comparing the time period September 2007–January 2006 to the time period February 2009–July 2010, the most noticeable changes were: 1) an increase in proportion of mothers that received the combination AZT+sdNVP – from 24.5% to 36.2%, and 2) a decrease in proportion of mothers that received sdNVP only – from 18.0% to 10.6%.

### Overall frequency of positive PCR

Of the 28,320 PCR tests performed, 3,481 (12.2%) were positive and 4 (<0.1%) were indeterminate. The overall percentages of positive PCR in age groups zero to six weeks, six weeks to six months and six to 12 months were 7.1%, 11.4% and 20.5% respectively.

### Frequency of positive PCR by those who received ARVs and type of ARV regimen

Across each age group, the rate of positive PCR varied with 1) whether the mother and/or the child received ARV drugs and 2) the type of ARV regimen received by the mother. Tables 2 and 3 provide details on the transmission rates and the 95% confidence intervals by age groups.

**Table 2:** Rate of positive PCR by “who received ARV drugs”, mothers' ARV regimen, mothers' age at the time of delivery, HIV status disclosure, and by children's age group.

Rate of PCR+ by “who received ARVs” and children age group						
Children age groups	Who received ARVs?	N	Children with PCR+	Transmission rate	95% CI	P-value
0–6 weeks	Both	4,827	280	5.8%	(5.1%–6.5%	<0.001
	Mother only	446	47	10.5%	(7.7%–13.4%	
	Baby only	70	11	15.7%	(6.9%–24.5%)	
	None	261	57	21.8%	(16.8%–26.9%)	
6 weeks–6 months	Both	11,918	958	8.0%	(7.6%–8.5%)	<0.001
	Mother only	1,.502	189	12.6%	(10.9%–14.3%)	
	Baby only	265	46	17.4%	(12.7%–22.0%)	
	None	1,.538	479	31.1%	(28.8%–33.5%)	
6–12 Months	Both	3,201	442	13.8%	(12.6%–15.0%)	<0.001
	Mother only	561	128	22.8%	(19.3%–26.3%)	
	Baby only	109	25	22.9%	(15.2%–30.7%)	
	None	1,182	428	36.2%	(33.5%–38.9%)	

Rate of PCR+ by mothers' ARV regimen and children age group						
Children age groups	Mother's ARV regimen	N	Children with PCR+	Transmission rate	95% CI	P-value
0–6 weeks	No Intervention	354	71	20.1%	(15.8%–24.3%)	<0.001
	NVP	1,143	100	8.7%	(7.1%–10.4%)	
	AZT+sdNVP	2,366	162	6.8%	(5.8%–7.9%)	
	HAART	1,966	82	4.2%	(3.3%–5.1%)	
6 weeks–6 months	No Intervention	1,894	555	29.3%	(27.3%–31.3%	<0.001
	NVP	3,263	441	13.5%	(12.3%–14.7%)	
	AZT+sdNVP	5,576	530	9.5%	(8.7%–10.3%)	
	HAART	5,141	241	4.7%	(4.1%–5.3%)	
6–12 Months	No Intervention	1,367	475	34.7%	(32.2%–37.3%	<0.001
	NVP	1,075	214	19.9%	(17.5%–22.3%)	
	AZT+sdNVP	1,255	193	15.4%	(13.4%–17.4%)	
	HAART	1,591	188	11.8%	(10.2%–13.4%)	

Rate of PCR+ by mother's age and children age group						
Children age groups	Mother's age at delivery	N	Children with PCR+	Transmission rate	95% CI	P-value
0–6 weeks	Mother<30	3,183	205	6.4%	(5.6%–7.3%)	0.010
	Mother≥30	1,519	70	4.6%	(3.6%–5.7%)	
6 weeks–6 months	Mother<30	7,821	692	8.8%	(8.2%–9.5%)	<0.001
	Mother≥30	3,770	228	6.0%	(5.3%–6.8%)	
6–12 months	Mother<30	1,981	304	15.3%	(13.8%–16.9%)	0.002
	Mother≥30	1,084	122	11.3%	(9.4%–13.1%)	

Rate of PCR+ by disclosure status and children age group						
Children age groups	Disclosure of HIV status	N	Children with PCR+	Transmission rate	95% CI	P-value
0–6 weeks	Didn't disclose	260	25	9.6%	(6.0%–13.2%)	0.115
	Disclosed	5,777	406	7.0%	(6.4%–7.7%)	
6 weeks–6 months	Didn't disclose	996	16	17.0%	(14.6%–19.3%)	<0.001
	Disclosed	15,548	1,709	11.0%	(10.5%–11.5%)	
6–12 months	Didn't disclose	525	135	25.7%	(22.0%–29.4%)	0.003
	Disclosed	5,107	1,021	20.0%	(18.9%–21.1%)	

**Table 3:** Rate of positive PCR by feeding mode, place and type of delivery, and by children's age group.

Rate of PCR+ by infant feeding mode, and children age group						
Children age groups	Feeding mode	N	Children with PCR+	Transmission rate	95% CI	P-value
0–6 weeks	Never breastfed	869	22	2.5%	(1.5%–3.6%)	<0.001
	Still BF*	3,815	247	6.5%	(5.7%–7.3%)	
	Stopped BF**	142	11	7.7%	(3.2%–12.2%)	
6 weeks–6 months	Never breastfed	1,899	82	4.3%	(3.4%–5.2%)	<0.001
	Still BF*	9,320	802	8.6%	(8.0%–9.2%)	
	Stopped BF**	699	74	10.6%	(8.3%–12.9%)	
6–12 months	Never breastfed	438	23	5.3%	(3.2%–7.3%)	<0.001
	Still BF*	1,351	226	16.7%	(14.7%–18.7%)	
	Stopped BF**	1,412	193	13.7%	(11.9%–15.5%)	

Rate of PCR+ by place of delivery and children age group						
Children age groups	Place of delivery	N	Children with PCR+	Transmission rate	95% CI	P-value
0–6 weeks	Home	573	74	12.9%	(10.2%–15.7%)	<0.001
	HF by C-section	257	9	3.5%	(1.2%–5.8%)	
	HF	5,172	348	6.7%	(6.0%–7.4%)	
6 weeks–6 months	Home	2,151	390	18.1%	(16.5%–19.8%)	<0.001
	HF by C-section	714	55	7.7%	(5.7%–9.7%)	
	HF	13,592	1,418	10.4%	(9.9%–10.9%)	
6–12 months	Home	950	245	25.8%	(23.0%–28.6%)	0.008
	HF by C-section	184	31	16.8%	(11.4%–22.3%)	
	HF	4.469	869	19.4%	(18.3%–20.6%)	

* This means the child was still breastfeeding at the time of dried blood spot (DBS) collection for PCR.

** This means the child was breastfed but stopped by the time of DBS collection. The timing when breastfeeding was stopped was not specified.

### Frequency of positive PCR by place and type of delivery

Across all age groups, the rate of positive PCR results was significantly highest when the child was delivered at home, followed by when delivered by C-section at health facility (HF), and lowest when children were delivered vaginally at health facility. For example, in age groups zero to six weeks, when born vaginally at home, vaginally at a health facility, or by C-section at health facility, the rate of positive PCR results was 12.9%, 6.7% and 3.5% respectively, (p-value<0.001).

### Frequency of positive PCR by mother age

Across all age groups, when both mother and infant received ARVs, the rate of positive PCR results was significantly lower when the child was born to a mother who is 30 year or older (Table 2).

### Frequency of positive PCR by disclosure of HIV-positive status

The rate of a child's positive PCR result was lower when mothers disclosed their HIV-positive to their partners, as compared to when they did not (Table 2). The difference was significant in the age groups of six weeks to 6 months and 6 months to 12 months. This remains true whether infants or mother only received ARVs or when both mother and infants received an ARV regimen.

#### Comparing ARV exposures and non ARV-related parameters

Estimating Adjusted Odds Ratio (95% Confidence Intervals) for ARV exposures and non ARV-related parameters we found that across all ages, the lowest rate of positive PCR test results was associated with 1) both mother and infant or mother alone received ARVs versus neither, 2) children never breastfed versus children still breastfeeding at the time of DBS collection, and 3) mother being 30 or older (Table 4).

**Table 4:** Estimated Adjusted Odds Ratio and 95% Confidence Intervals for ARV exposures and non ARV-related parameters.

PMTCT interventions or characteristic of population	Age Group		
	0–6 weeks	6 weeks–6 months	6–12 months
**Who received ARV?**			
Both	0.30 (0.21–0.44)	0.21 (0.18–0.24)	0.30 (0.25–0.36)
Mother only	0.47 (0.30–0.74)	0.33 (0.27–0.40)	0.55 (0.43–0.70)
Baby only	0.87 (0.41–1.81)	0.50 (0.36–0.71)	0.53 (0.32–0.85)
Neither	1	1	1
**Feeding method?**			
Never breastfed	0.45 (0.30–0.70)	0.47 (0.38–0.59)	0.27 (0.19–0.40)
Still breastfeeding	1	1	1
**Place and type of delivery?**			
Home	1.35 (0.96–1.90)	1.05 (0.90–1.23)	0.86 (0.70–1.06)
Health Facility, C-section	0.77 (0.37–1.60)	0.92 (0.67–1.27)	1.04 (0.67–1.61)
Health facility, no C-section	1	1	1
**Maternal age?**			
Less than 30 years old	1.47 (1.15–1.89)	1.31 (1.16–1.47)	1.35 (1.15–1.58)
At least 30 years old	1	1	1
**Disclosure status?**			
Did not disclose	1.26 (0.79–2.00)	1.11 (0.90–1.36)	1.06 (0.82–1.37)
Disclosed	1	1	1

#### Frequency of positive PCR rates overtime

Between September 2007 and January 2009, and between February 2009 and July 2010, the overall proportions of positive PCR were 15.1% and 11% respectively.

By age group, regardless of whether infants received ARVs or not, when mothers received ARV drugs, the proportion of positive PCR results was lower in the later of the time periods mentioned above. Differences were significant in the age groups 6weeks–6 months and 6–12 months. (Table 5).

**Table 5:** Rate of positive PCR by time period of DBS collection and by children's age group.

Children age groups	Time period	N	Children with PCR+	Transmission rate	95% CI
0–6 weeks	Sep 07–Jan 09	1,502	109	7.3%	(5.9%–8.6%)
	Feb 09–July 10	4,026	237	5.9%	(5.2%–6.6%)
6 weeks–6 months	Sep 07–Jan 09	4,169	438	10.5%	(9.6%–11.4%)
	Feb 09–July 10	9,914	785	7.9%	(7.4%–8.4%)
6–12 Months	Sep 07–Jan 09	1,393	241	17.3%	(15.3%–19.3%)
	Feb 09–July 10	2,556	357	14.0%	(12.6%–15.3%)

## Discussion

Analysis of data from PMTCT program implementation showed a reduced risk of MTCT (3.5 to 12.9%) compared to when to when ARVs are not provided (15 to 45%) [10]. Our finding is comparable to those in many other publications [11], [12], [13], [14]. We had a similar finding when we previously analyzed a smaller subset of the same data presented here [6], [7].

Compared to the previous analysis, we observed a lower rate of MTCT [6]. (Table 5. This change is more likely an indication that PMTCT programs were better implemented over time. Between September 2007 and July 2010, the proportions of 1) mothers accessing efficacious regimens had increased; and 2) mothers and infants receiving no ARVs or sdNVP only had decreased. (Table 1).

In 2000, HIVNET and SAINT studies found a six week MTCT rate of 11.8% and 12.3% respectively using sdNVP compared to 8.7% in our study [15], [16]. Unlike these studies that reviewed a sdNVP only regimen, this papers presents the analysis of a mixture of the sdNVP only regimen and other more efficacious ARV regimens. Our results show the rate of MTCT in the group that received an AZT-based ARV regimen was similar to the MTCT rates found in the DITRAME Plus study in Cote D'Ivoire [17]. The overall MTCT rate in our analysis was also affected by the substantial proportion of mothers and/or infants that did not actually receive ARVs. The majority of these were in the early phase of the early infant diagnosis program where access to PMTCT services was limited.

Although in our population, breastfeeding was the common feeding method, the rates of MTCT that were observed compare with MTCT rates observed in clinical trials where the majority of babies were predominantly formula-fed. For example, Creek et al in Botswana found a 5% MTCT rate at eight weeks postnatal [18]. In our analysis, MTCT rates vary between 4.2% (when mothers received HAART) and 8.7% (when mothers received sdNVP only.

The major contribution of our study is to show how analysis of routine program data can be a useful tool for assessing the outcome of a PMTCT program and by doing so, help adjust or fine-tune program implementation in an evidence-informed way. Our analysis highlighted how MTCT is significantly reduced when ARVs are used. Furthermore, it showed how the use of ARV regimens that maximally suppress viral replication can reduce the risk of MTCT. The use of ARVs remains the cornerstone of PMTCT interventions and combined ARVs were associated with even greater reduction of MTCT. Indeed we observed better PMTCT outcomes when mother initiated lifelong ART or other combined ARVs than when sdNVP was the only regimen implemented. This is a call for programs to strive for implementing the most highly efficacious regimen possible [9], [19].

ARV based interventions, although critical, are only part of PMTCT programs. Our analysis unveiled how non-ARV related interventions were also associated with the rate of MTCT in the target population. The age of mothers at the time of delivery was associated with the rate of MTCT observed.

We found an increment of the overall MTCT rate between the zero to six weeks and six months to 12 months age group. We observed that the increment of crude MTCT rates between the zero to six weeks age group and six weeks to six months group was 4.3%, comparable to 4.5% increment found by the Mashi study [20]. The increasing rate can be attributed to postnatal transmission.

Furthermore, our data showed that 52.8% of the mothers were still breastfeeding (including mixed feeding) after 12 months. This is a call for PMTCT programs to urgently implement interventions that reduce risk of MTCT during the breastfeeding period [9], [21], [22], [23]. This recommendation is further supported by a pooled analysis of five randomized controlled trials which employed extended use of nevirapine and AZT during the breastfeeding period, which found a 70% reduction in the MTCT rate [20]. In our study population, replacement feeding is usually not an option because of acceptability, feasibility, affordability, safety and sustainability concerns [24]. The WHO Update on ARV options for PMTCT comes timely [25]. In addition to other ARVs regimens, countries like Zambia have a larger choice including lifelong triple ARV combination (or “option B+”). More HIV-positive mothers are covered by ARV therapy, especially during breastfeeding period to reduce the risk of MTCT in postnatal period.

In the univariate analysis, we observed that higher rate of MTCT was associated with 1) mother and newborn not receiving ARVs, 2) home delivery, 3) not breastfeeding, 4) being born to a mother younger than 30 years of age, and 5) mother not disclosing her HIV-status. The association was significant in the age groups six weeks to six months and six months to 12 months. In the multivariate analysis, of the above five parameters adjusted for ARV regimens, only 1) mother and newborn not receiving ARVs, 2) breastfeeding and 3) being born to a mother younger than 30 years of age were associated with the highest MTCT rates. The association noticed here was stronger compared to our previous analysis [7]. This is more likely due to the smaller sample size of the previous analysis.

Although there is no plausible direct causal link, we believe that each of the three parameters – place of delivery, disclosure of HIV positive status, and mother's age – do influence adherence to ARVs regimen, infant feeding practices, or counseling offered during PMTCT interventions. Several studies have found that male partners or family influence women's choices about infant feeding methods or adherence to an ARV regimen [26], [27], [28], [29]. Mother's age or whether she had disclosed her HIV-positive status may determine how her partner or family would influence her infant feeding choice and adherence. In our analysis, we found that the influence of HIV-status disclosure on MTCT rate was significant after the 6^th^ week postnatal period, which is a time when the infant feeding method affects the mother–to-child transmission of HIV. These results suggest that a critical look of the non-medical aspects of PMTCT implementation be taken. For example, mothers younger than 30 years might need more adherence or infant feeding counseling than older mothers. Additionally, efforts to increase adherence to PMTCT regimens should incorporate support for disclosing HIV status to the partners when it does not put the mothers' life in danger.

Our study had also contributed to the performance and quality of PMTCT in the five provinces where these data come from. It provided an opportunity to analyze PMTCT activities, study the provision and immediate outcomes of services and consequently improve them.

Our study has similar limitations to those described in our previous analysis [6], [7]. HIV exposed children that did not show up for EID might be the very ones that were infected and/or died. These children were counted neither in the numerator nor the denominator, thus the MTCT rate that our study reports might be an underestimation of the true MTCT rate.

Furthermore, given the repeated cross-sectional nature of our data, we are unable to distinguish any effects of age from cohort or history effects; for example, the effect of age is masked with any changes in PMTCT regimens that occurred over time. In addition, using observational data precludes making strong causal statements when comparing estimates of transmission rates.

## Conclusion

The use of ARV drugs reduces vertical transmission of HIV in a program setting. However non chemoprophylactic factors especially breastfeeding has a significant effect on postnatal HIV transmission. The overall change in the proportions of positive PCR over time is more likely an indication of improved PMTCT implementation. Determination of the impact of these programmatic interventions is feasible and requires accurate documentation and analysis.
